# Incidence and risk factors for contrast-induced nephropathy after angioplasty: an observational retrospective study

**DOI:** 10.1590/1677-5449.202500282

**Published:** 2026-01-09

**Authors:** Juliana Peres, Jeferson Freitas Toregeani, Amanda Cristina Pohl, Ana Julia Vendrametto, Luciano de Andrade, André Brusamolin Moro

**Affiliations:** 1 Centro Universitário Fundação Assis Gurgacz – FAG, Cascavel, PR, Brasil.; 2 Universidade Estadual do Oeste do Paraná – UNIOESTE, Cascavel, PR, Brasil.; 3 Universidade Estadual de Maringá – UEM, Maringá, PR, Brasil.; 4 Hospital Estadual Vila Alpina, São Paulo, SP, Brasil.

**Keywords:** angioplasty, contrast media, acute kidney injury, kidney diseases, risk factors

## Abstract

**Background:**

Contrast-induced nephropathy (CIN) is a complication of iodinated contrast media use that can lead to worsening of renal function, increased morbidity and mortality, and the need for renal replacement therapy.

**Objectives:**

To evaluate the incidence of CIN after angioplasty and identify associated factors, including variations in creatinine, glomerular filtration rate (GFR), contrast volume, procedure time, and comorbidities.

**Methods:**

Retrospective study of 305 patients undergoing angioplasty. Clinical and laboratory variables, including serum creatinine and GFR before and after the procedure, were analyzed. CIN was defined as an absolute increase in creatinine ≥ 0.3 mg/dL or a relative increase ≥ 50% within 48 hours. Logistic regression was applied to identify independent predictors.

**Results:**

The incidence of CIN was 10.5% (n = 32/305). Patients with CIN showed a significant reduction in GFR (pre: 73.79 ± 22.5 vs. post: 34.32 ± 11.8 mL/min; p < 0.0001) and increased creatinine (pre: 1.12 ± 0.3 vs. post: 1.78 ± 0.6 mg/dL; p < 0.001). CIN was associated with stroke (p = 0.014), peripheral arterial occlusive disease (p = 0.007), diabetes mellitus (p = 0.002), chronic kidney disease (p = 0.005), and heart failure (p = 0.004). Multivariate analysis confirmed DM (OR = 2.45; 95% CI: 1.12–4.38; p = 0.022) as the main risk factor.

**Conclusions:**

CIN occurred in 10.5% of patients, with DM, CKD, and HF being the main risk factors. These findings reinforce the importance of monitoring to reduce the impact of CIN and optimize clinical outcomes.

## INTRODUCTION

Contrast-induced nephropathy (CIN) is a significant complication seen in individuals who have undergone angioplasty procedures and one that is frequently underdiagnosed and underestimated.^1^ Angiography is a common technique in cardiovascular and vascular surgery that uses X-rays to enable visualization of arteries and veins, generally using iodinated contrasts to enhance the vascular images.^2,3^ Despite advances in the formulations of low osmolality contrast media, which are considered less harmful,^4,5^ CIN remains the third greatest cause of acute renal failure in hospital settings, accounting for around 10% of cases.^6^ The condition causes increased length of hospital stay, need for intensive care, and increased hospital expenses, including the cost of medications and dialysis treatments. Moreover, CIN increases readmission rates and delays patient recovery, with a direct impact on morbidity and mortality.^7^

While CIN is a growing concern in clinical practice, there are still many risk factors that need to be explored in depth, including epidemiological characteristics, duration of contrast exposure, and associated comorbidities.^8^ The current literature contains a variety of data and lessons about these factors that are very often contradictory, especially with regard to the safe contrast volume cutoff point, the true influence associated comorbidities have on the patient in terms of CIN incidence, and the preventive effectiveness of hydration strategies.^4^ While the volume of contrast has traditionally been associated with CIN,^1,6,8^ recent studies have reported conflicting results, varying from a strong association to an absence of any causal relationship,^9,10^ highlighting the need for more rigorous protocols for optimization of the dose administered. This scenario underscores the importance of a more comprehensive investigation.^11^

CIN is characterized by rapid changes in renal function after administration of iodinated contrast media, defined as an absolute increase in serum creatinine of ≥ 0.5 mg/dL or a relative increase of ≥ 25% up to 72 hours after infusion of contrast.^12-14^ The etiopathogenesis of CIN is multifactorial, in which vasoconstriction is one of the most important mechanisms, causing renal medullary ischemia and reducing the glomerular filtration rate (GFR).^2,6^ Additionally, the pharmacotoxic effects of contrast media can also cause direct cell damage, interference with renal tubules, and osmotic changes, provoking a toxic effect regardless of hemodynamic changes.^15^ Iodinated contrasts can provoke increases in sodium concentration and osmotic pressure in the renal tubule, contributing to acute tubular injury and further compromising renal function.^16^

Risk factors for CIN include preexisting conditions that impair renal function, such as chronic renal failure (CRF), diabetes mellitus (DM), advanced age, heart failure (HF), dyslipidemia, and systemic arterial hypertension (SAH).^17-19^ Additionally, characteristics of the contrast medium such as the volume administered and its osmolality also affect the risk,^13,20,21^ with higher osmolality contrasts more likely to increase osmotic pressure and intensify vasoconstriction and renal toxicity.^4,11^ The choice of contrast should therefore take these factors into account to reduce the risk of CIN.

Laboratory assessment is essential for patients undergoing angioplasty who are at risk of developing CIN, primarily because of the complexity of the disease.^11^ Serum creatinine assay is one of the main methods used to detect changes in renal function. Studies indicate that elevation of creatinine levels after exposure to contrast may be an effective predictor of acute kidney damage, enabling early and appropriate intervention.^22^ However, more comprehensive studies indicate that calculating the GFR is a more precise and reliable method for assessing renal function. A GFR assessment is essential for identifying individuals at elevated risk of CIN, especially those with CRF, DM, and SAH. These risk factors are robustly associated with development of CIN, highlighting the importance of prior and continuous renal assessment in patients who will undergo procedures with contrast.^23,24^ Moreover, additional laboratory markers are recommended for a complete assessment of renal function.^25^

Laboratory monitoring should be supplemented by a comprehensive clinical assessment, taking account of factors such as age, comorbidities, and total volume of contrast medium administered as a function of the patient’s weight. Personalized management based on each patient’s characteristics and laboratory results is essential to reduce the impact of CIN and improve clinical outcomes.^26^ The evidence indicates that scientific training and raising awareness among health professionals of the risks of iodinated contrasts, in conjunction with rigorous laboratory assessments, are crucial strategies for prevention of CIN.^27,28^

The objective of this study was to investigate the incidence of CIN among patients who underwent angioplasty procedures at a hospital in the West of Paraná state (Brazil) and identify the risk factors associated with this outcome, providing evidence on the importance of clinical and laboratory monitoring for a minimum of 48 hours after the procedure.

## METHODS

### Study type and setting

This retrospective observational study was approved by the Human Research Ethics Committee at the Centro Universitário Fundação Assis Gurgacz (FAG) under Ethics Appraisal Submission Certificate number 80063724.0.0000.5219 and consolidated opinion number 6.949.781. It was conducted over an 8-month period with the objective of analyzing the patient medical records of people who underwent angioplasty with contrast at a hospital in the West of Paraná. Considering its retrospective nature and the large population involved in the study, a free and informed consent form waiver was granted by the ethics committee.

### Study population

This study employed a consecutive selection method, including all patients who underwent angioplasty with non-ionic iodinated contrast from 2021 to 2024 at a teaching hospital in the West of Paraná. For standardization, the same intravenous contrast medium (Omnipaque®, Ioexol 300 mg/mL, 600-844 mOsm/Kg/H2O) was used in all cases.

Patients were excluded from the study if they were under the age of 18, had had prior exposure to iodinated contrast during the 30 days preceding inclusion in the study, had died less than 48 hours after administration of the contrast, or if insufficient preoperative or postoperative laboratory or epidemiological data were available for the analyses.

To minimize the risk of selection bias, the inclusion and exclusion criteria were standardized, guaranteeing the representativeness of the sample, without subjective interference. Exclusions were limited to cases that could compromise the analysis of CIN, avoiding distortion of the results. Standardization of the contrast medium eliminated variations in renal toxicity, while the objective definition of CIN adopted prevented misclassification errors. Analysis with multivariate logistic regression enabled control of confounding factors, making the analysis more precise and reliable.

**Figure 1:**
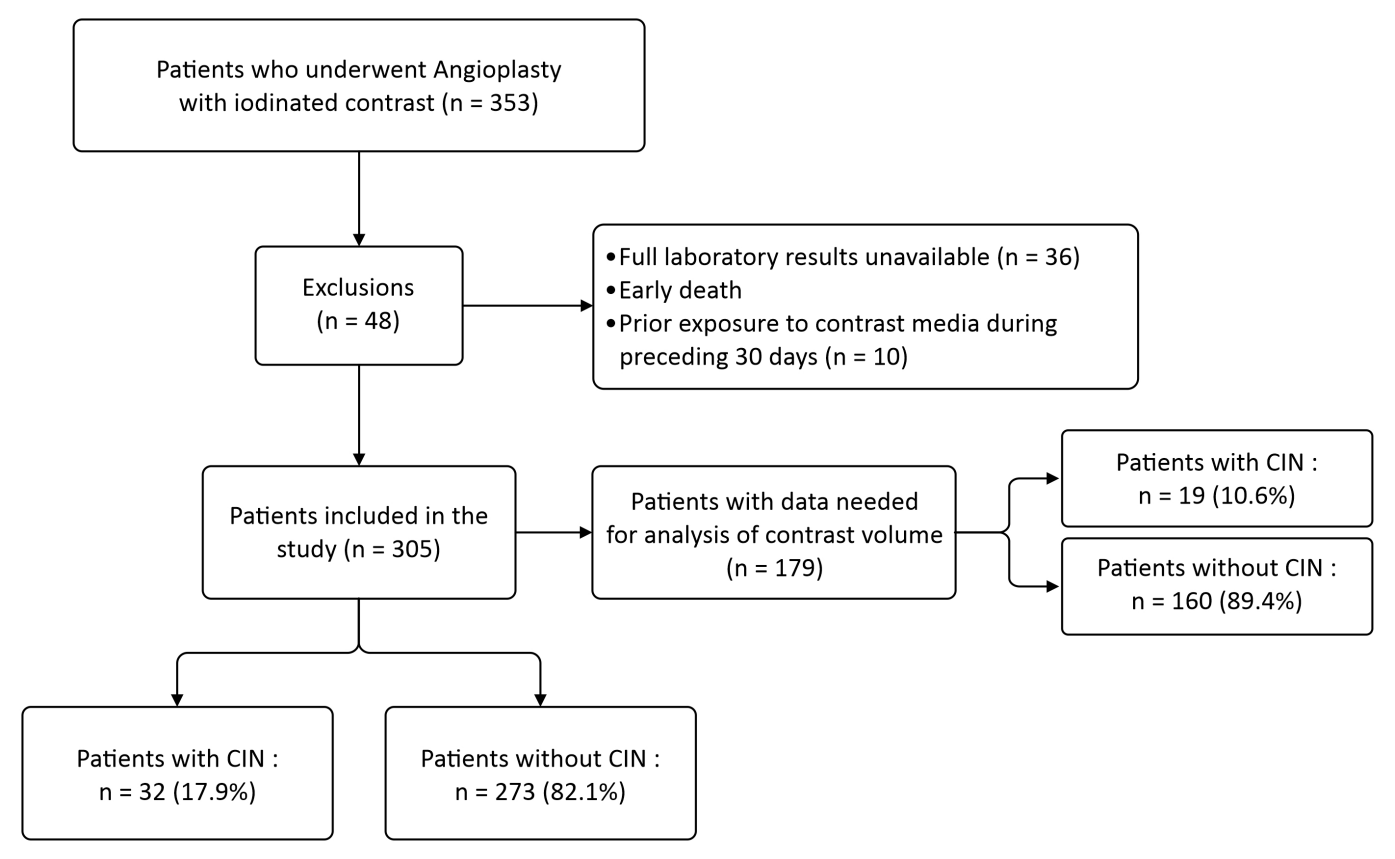
Flow diagram illustrating the process of selection, exclusion, and losses of study participants. CIN = contrast-induced nephropathy.

Overall, a total of 353 individuals were selected, 48 of whom were later excluded. Thirty-six of these did not have sufficient preoperative or postoperative laboratory test results available to determine whether they had developed CIN, two had died 24 hours after hospital admission, and 10 had undergone a previous procedure involving exposure to iodinated contrast during the preceding 30 days. Although the total study sample was 305 patients, it was only possible to obtain full data on contrast volume from 179 patient medical records. The non-availability of this variable was because the exact volume had not been recorded in some of the electronic patient records, which made it impossible to include them in the statistical analysis. Therefore, any analyses that involved this variable were conducted using exclusively those with data available, guaranteeing that the analyses were conducted in a transparent manner and without imputation bias (Figure 1).

To guarantee sufficient statistical power, the minimum sample size was calculated as 109 participants, based on a formula for finite populations presented in Equation 1 (considering an incidence of CIN = 12.5%, n = 305, 95%CI, and error = 5%):


$$\begin{array}{c} n\;=\;\left[305\;x\;0.125\;x\;\left(1\;-\;0.124\right)\;x\;\left(1.96\right)²\right]\;/\;\\ \left\{\left[\left(305\;-\;1\right)\;x\;\left(0.05\right)²\right]\;+\;\left[0.125\;x\;\left(1\;-\;0.125\right)\;x\;\left(1.96\right)²\right]\right\} \end{array}$$ (1)

However, it was decided to include all 305 eligible cases to increase the precision of estimates.

### Variables analyzed

The variables collected from the electronic patient records were sex, age, weight, ethnicity, preexisting comorbidities, preoperative and postoperative serum creatinine levels (up to 72 hours after the procedure), procedure performed, contrast volume administered, and procedure time. Preoperative and postoperative GFR was calculated using the Cockcroft-Gault equation.^29^

### Primary outcome

CIN after angioplasty procedures, whether elective or emergency, was the primary outcome analyzed in the study. CIN was defined as rapid deterioration in renal function after administration of iodinated contrasts, diagnosed as an absolute increase in serum creatinine of ≥ 0.5 mg/dL or a relative increase of ≥ 25% up to 72 hours after infusion of the contrast.^12,13^ The study’s secondary objective was to assess the incidence of CIN and identify risk factors associated with it, with special attention to patients’ preexisting comorbidities.

### Data analysis

The data collected were synthesized and organized in an Excel® spreadsheet and then analyzed descriptively. Continuous variables (age, weight, preoperative and postoperative creatinine, contrast volume infused, and preoperative and postoperative GFR) were expressed as mean ± standard deviation for normal distributions and as median and interquartile range for non-normal distributions. Categorical variables were expressed as absolute frequencies and percentages. Comparisons between groups (patients with or without CIN) for continuous variables were conducted using the Mann-Whitney (nonparametric) test, because of the non-normal distributions. Categorical variables were compared using the chi-square test or Fisher’s exact test, as appropriate. Factors associated with development of CIN were identified using multivariate logistic regression analysis, employing the backward stepwise method and the Wald statistic. Odds ratios (OR) and 95%CIs were presented to quantify the magnitude of associations. P values < 0.05 were considered statistically significant for all analyses.

Appropriate nonparametric statistical tests were used for analyses involving contrast volume, because of the non-normal distribution. Comparisons between groups with and without CIN were conducted using the Mann-Whitney test, while the association between contrast volume and procedure time was investigated using Spearman’s correlation. The influence of procedure type on contrast volume was analyzed using the Kruskal-Wallis test.

All data were analyzed using GraphPad Prism statistical software. version 10.3.9. Backward stepwise logistic regression was used to investigate correlations between independent variables and development of CIN and this analysis was performed using the Statistical Package for the Social Sciences® (version 17.0 for Windows). Additionally, this study followed the recommendations of the STROBE Statement for observational studies, as detailed in the Supplementary Material – STROBE Statement provided in the online version of the article.

## RESULTS

The sample included in the study comprised 305 individuals. There were 197 (64.6%) men and 108 (35.4%) women, including 190 white-skinned patients (62.3%) and 115 (37.7%) brown or black-skinned patients. The age of the participants ranged from 28 to 90 years, with a mean of 66.69 ± 11.02 years and median of 68 years. Body weight ranged from 30 to 168 kg, with a mean of 76.52 ± 17.40 kg and median of 74 kg. Mean preoperative serum creatinine was 1.07 ± 0.54 mg/dL and median was 0.93 mg/dL, while mean postoperative creatinine was 1.21 ± 0.77 mg/dL and median was 1.05 mg/dL. Preoperative GFR ranged from 13.4 to 252.8 mL/min, with a mean of 80.2 ± 36.2 mL/min, and postoperative GFR ranged from 10.5 to 288.2 mL/min, with a mean of 74.0 ± 35.9 mL/min. The volume of contrast infused ranged from 2 to 600 mL, with a mean of 170 ± 99.0 mL and median of 150 mL. Correction by body surface area gave a value of 89.9 ± 52.4 mL/m^2^. The incidence of CIN was 10.5%.

The Mann-Whitney test was applied to the variables mean procedure time and anatomic site of angioplasty. The results are shown in Table 1.

**Table 1:** Angioplasty site and mean duration of procedures.

**Site**	**n total**	**Mean time**	**n with CIN**	**Mean time**
Angioplasty of coronary arteries	244	01:21 ± 00:48	20	01:34 ± 01:11
				p = 0.36
Angioplasty of renal arteries	2	01:41 ± 00:12	1	01:32
Endovascular treatment of aortic diseases	8	03:15 ± 00:34	2	03:16 ± 00:07
				p = 0.57
Angioplasty of carotid arteries	25	01:54 ± 01:01	4	01:18 ± 00:22
				p = 0.37
Angioplasty of the subclavian artery	2	00:59 ± 00:36	1	00:33
Angioplasty of the lower limbs	23	01:42 ± 00:48	4	01:11 ± 00:37
				p = 0.19
Angioplasty of the supra-aortic trunk	1	01:01	-	-
Total	305	01:28	32	01:33 ± 01:04
				p = 0.63

CIN = contrast-induced nephropathy.

Source: study data.

Comparison of patients who did or did not have CIN showed that those who did develop CIN had a slightly higher mean age (69.5 years vs. 68.0 years; p = 0.95). Weight also did not exhibit any relevant difference between these two groups (77.0 kg vs. 74.0 kg; p = 0.98). As expected, there was a trend for individuals with CIN to have a lower preoperative GFR than those in the group without CIN (61.08 mL/min vs. 77.78 mL/min; p = 0.09), but the difference was not statistically significant. Notwithstanding, postoperative GFR was significantly lower in the group with CIN (34.32 mL/min vs. 73.79 mL/min; p < 0.0001), indicating a deterioration in renal function after the procedure among these patients, as required for the diagnosis. Additionally, preoperative creatinine was higher among individuals with CIN (1.1 mg/dL vs. 0.92 mg/dL; p = 0.03) and the postoperative elevation of creatinine was even more pronounced, with a mean increase of 2.1 mg/dL among patients with CIN, compared with 1.00 mg/dL among individuals without CIN (p < 0.0001). None of the remaining comparisons of demographic, clinical, or laboratory variables identified significant differences between patients with and without CIN, as shown in Table 2.

**Table 2:** Comparison of continuous variables between individuals with and without CIN.

	**Without CIN (n = 273)**	**With CIN (n = 32)**	**P**
Age (years)	68.00 ± 11.00	69.50 ± 10.37	0.95
Weight (kg)	74.00 ± 17.31	77.00 ± 18.34	0.98
Preoperative GFR (mL/min)	77.70 ± 35.46	61.08 ± 41.32	0.09
Postoperative GFR (mL/min)	73.79 ± 34.95	34.32 ± 19.76	< 0.0001
Preoperative creatinine (mg/dL)	0.92 ± 0.40	1.10 ± 1.08	0.03
Postoperative creatinine (mg/dL)	1.00 ± 0.39	2.10 ± 1.50	< 0.0001

GFR = glomerular filtration rate; CIN = contrast-induced nephropathy.

Source: study data.

Analysis of relationships between comorbidities and incidence of CIN (Table 3) revealed important associations between certain clinical conditions and increased risk of CIN. Comorbidities such as stroke, peripheral arterial occlusive disease (PAOD), DM, HF, and CRF all exhibited significant p values (< 0.05), demonstrating that individuals with these conditions have a greater likelihood of developing the disease. DM and chronic kidney disease (CKD) stood out, with ORs of 3.1 and 20.77 respectively. Additionally, HF had an OR of 3.1 (95%CI 1.2-8.0), underscoring its role in increased risk of CIN. In contrast, although comorbidities such as SAH and dyslipidemia were common among these patients, they did not have significant associations, as indicated by their p values > 0.05 and ORs close to 1, indicated that these conditions, in isolation, do not increase the risk of CIN. However, the prevalence of SAH among patients with CIN (71.88%) suggests that, while it was not a risk factor in isolation, it could play a role in conjunction with other comorbidities.

**Table 3:** Comparison of comorbidities with incidence of CIN.

**Comorbidities**	**p (< 0.05)**	**OR**	**95%CI**
Stroke	0.004	4.40	1.6-12.8
CAD	0.70	0.80	0.3-2.1
PAOD	0.02	3.40	1.2-10.1
Dyslipidemia	0.14	0.50	0.2-1.1
DM	0.002	3.10	1.5-6.5
SAH	0.20	0.60	0.2-1.5
Hypothyroidism	0.69	1.20	0.4-3.7
AMI	0.79	1.10	0.4-3.4
HF	0.01	3.10	1.2-8.0
CKD	< 0.0001	20.77	5.4-77.5
CVI	0.10	3.57	0.6-17.8
Smoking	0.10	1.80	0.8-3.8

CIN = contrast-induced nephropathy; CAD = chronic arterial disease; PAOD = peripheral arterial occlusive disease; DM = diabetes mellitus; SAH = systemic arterial hypertension; AMI = acute myocardial infarction; HF = heart failure; CKD = chronic kidney disease; CVI = chronic venous insufficiency; OR = odds ratio.

Source: study data AMI.

Analysis of the contrast volume infused in the groups with and without CIN did not reveal a statistically significant difference, according to the Mann-Whitney test. The group without CIN were given a mean volume of 174 ± 115 mL, whereas the group that did develop CIN received 183 ± 109 mL (p = 0.619), suggesting that, in isolation, the contrast volume infused was not a determinant factor of CIN in this sample.

Backward stepwise logistic regression was used to analyze variables such as sex, age, weight, ethnicity, procedure time, preoperative and postoperative GFR, and preoperative and postoperative creatinine. There was a positive correlation between CIN and postoperative creatinine values (p < 0.001, Exp(B) = 17.581), indicating that risk of CIN increased as postoperative creatinine increased, as has been observed in the literature previously. The Exp(B) value represents the estimated odds ratio in the model, that is, it quantifies how much the odds of the outcome increase (or decrease) for each unit increase in the predictor variable, holding the others constant. In this case, an Exp(B) of 17.581 means that for each 1 mg/dL increase in postoperative creatinine, the odds of developing CIN were approximately 17.6 times higher. Table 4 shows the variables in the backward stepwise logistic regression model.

**Table 4:** Variables in backward stepwise logistic regression .

**Variable**	**Score**	**df**	**P**
Sex	4.828	1	0.028
Age (years)	1.079	1	0.299
Weight (kg)	0.001	1	0.974
Ethnicity	0.100	1	0.752
Procedure time (min)	0.188	1	0.664
Preoperative GFR	20.570	1	< 0.001
Preoperative creatinine	63.530	1	< 0.001

GFR = estimated glomerular filtration rate; df = degrees of freedom, i.e., the number of independent values that can vary in the statistical analysis.

Source: study data.

The Spearman test indicated that there was a positive correlation between contrast volume and procedure time, but this was not significant (ρ = 0.124; p = 0.098), suggesting that these variables were independent of each other. The Kruskal-Wallis test (chi-square = 4.24; p = 0.644) and Dwass-Steel-Critchlow-Fligner multiple comparisons (p > 0.05) did not identify significant differences in contrast volume between the different types of angioplasty.

Table 5 shows the results of chi-square tests used to assess the incidence of CIN stratified by contrast volume administered, revealing that although the group administered > 200 mL had a numerically greater incidence (20.0% vs. 10.1% in the ≤ 100 mL group), this difference was not statistically significant (p = 0.410). Nevertheless, the OR of 2.23 (95%CI 0.71-6.97) suggests that it possibly has clinical relevance, even without statistical significance.

**Table 5:** Analysis of the association between contrast volume ranges and CIN according to the chi-square test

**Range of volume infused (mL)**	**n**	**Cases with CIN (%)**	**Relative risk (95%CI)**	**P**
< 100	79	8 (10.1)	1.00 (Ref^*^)	-
101-200	70	8 (11.4)	1.13 (0.43-2.96)	0.814
> 200	30	6 (20.0)	1.98 (0.74-5.30)	0.108
Total	179	22 (12.3)		

* P values calculated with Fisher’s exact test (bilateral), comparing each range with the reference range (≤ 100 mL). P values of < 0.05 were considered statistically significant. CIN = contrast-induced nephropathy.

Source: study data.

## DISCUSSION

The analysis revealed a 10.5% incidence of CIN in a sample of 305 patients who underwent angioplasty procedures. This finding is in agreement with existing literature, which reports incidence ranging from 2 to 30%, depending on the characteristics of the patients and the type of contrast used.^13,30^ The observation that postoperative GFR was significantly reduced in individuals who developed CIN (34.32 mL/min vs. 73.79 mL/min; p < 0.0001) indicates and accentuated deterioration in renal function, demonstrating the severity of these CIN cases, which could cause patients to need dialysis and increase mortality among cases with GFR ≤ 45 mL/min/1.73 m^2^.^9^ These data suggest that careful assessment of renal function before and after administration of contrast is crucial, especially in patients with known risk factors, such as DM and CKD,^31,32^ which exhibited ORs of 3.1 and 20.77, respectively. These results corroborate the hypothesis that baseline renal function is a significant predictor of CIN, underscoring the need for intensive clinical monitoring in this risk group.^33^

Analysis of comorbidities revealed that stroke, PAOD, and HF were also associated with a significant increase in the risk of CIN, highlighting the complexity of patients who undergo angiographic interventions.^10^ Notably, the prevalence of SAH among patients with CIN (71.88%) suggests that, although it was not shown to be a risk factor in isolation, it could interact in a relevant manner with other comorbidities, increasing vulnerability to development of CIN.^11,34,35^ The interaction between multiple clinical conditions underscores the need for a multidisciplinary approach to risk assessment and implementation of renal protection strategies, such as adequate hydration and use of nephroprotective agents when indicated.^6,13,27^

There were no statistically significant differences in mean time taken for procedures between cases with and without CIN for any of the anatomic sites, as shown by the Mann-Whitney test (p > 0.05 for all comparisons). Mean procedure time varied considerably between different sites, with the greatest duration for endovascular treatments of diseases of the aorta. The absence of statistical significance suggests that procedure time, in isolation, may not be a determinant factor in development of CIN. However, this analysis is subject to biases, such as the small number of procedures involving certain sites, (for example, angioplasty of renal arteries and the supra-aortic trunk), limiting the precision of comparisons.^36^

The backward stepwise logistic regression identified postoperative creatinine as the principal marker of CIN, with an OR of 17.5, reaffirming previous studies that have emphasized the importance of monitoring creatinine after administration of contrast for up to 48 hours after the procedure.^37,38^ The positive correlation between elevated postoperative creatinine levels and development of CIN is a clear indication that early intervention in patients with changes to renal function could be vital to prevent additional complications.^13^ These results corroborate the need for clinical protocols that include real-time assessment of renal function during and after angioplasty procedures, to ensure early identification and intervention in patients at risk, with the objective of minimizing the morbidity associated with CIN.^8,32,39,40^ Considering the clinical implications discussed, it is evident that individualization of therapeutic management of patients who have undergone angioplasty should be based on in-depth comprehension of their individual comorbidities and risk factors.

Even though contrast volume is widely recognized as a risk factor for CIN, in the present study no statistically significant association was found between this variable and the renal outcome. This finding contrasts with previous studies that have demonstrated a dose-dependent relationship between contrast volume and renal function.^11,13^ However, this discrepancy can be explained by certain methodological and clinical factors.

First, the limited availability of information on the contrast volume administered in our sample (available for just 172 of the 305 patients) reduced the statistical power of the analysis, making it less likely that any possible association would be identified. Additionally, clinical practices such as adequate perioperative hydration and preferential use of contrasts with lower osmolality may have mitigated the nephrotoxic effects of the contrast, reducing its impact on renal function.^6,27,34^ There is evidence to suggest that hydration guided by individualized strategies may be more effective for prevention of CIN than simply restricting the volume of contrast used.^35^

Another relevant point is that CIN is a multifactorial phenomenon, influenced by preexisting conditions, such as DM, CRF, and HF, which are all factors that were shown to be significantly associated with occurrence of CIN in our analysis. Previous studies demonstrate that individual predisposition plays a more determinant role that contrast volume in isolation, especially in patients with low GFR.^15,19^ This could explain why, even in studies that have demonstrated an association between contrast volume and CIN, the thresholds of risk vary widely depending on patients’ characteristics.^16^

This study has certain limitations that should be considered. While the sample is representative, it may not completely reflect the angioplasty population, limiting the generalizability of the findings. Moreover, the analysis was conducted at a single center, which could introduce biases related to institutional practices and the specific profile of the patients cared for. The retrospective design also imposes challenges, such as possible imprecisions in physicians’ records and the absence of planned information on additional risk factors or renal protection strategies.^7^

Another relevant limitation is related to the absence of information on use of nephroprotective agents, such as statins and N-acetylcysteine, which could have influenced the renal outcomes.^5,18^ Additionally, variability in the treatment approach, including hydration time before and after the procedure, could have contributed to the heterogeneous results.

As such, future studies are needed to better elucidate the relationship between contrast volume and CIN. Studies with prospective designs, inclusion of control groups, and more comprehensive diagnostic criteria for renal damage could provide more robust evidence on the impact of contrast volume on renal function and identify subsets of patients with greater vulnerability to this effect.^20,24^

## CONCLUSIONS

Rigorous monitoring of renal function and assessment of comorbidities are essential to reduce the risk of CIN and improve clinical outcomes in patients who undergo angioplasty. In the present study, CKD and DM were identified as significant risk factors, highlighting the need for specific protocols for patients with these conditions. The high OR associated with CKD underscores its relevance as a critical determinant of CIN, demanding additional precautions to minimize kidney damage. Along the same lines, the significant association between DM and CIN illustrates the interaction between metabolic dysfunction and renal vulnerability, justifying different preventative approaches for this subset. As such, personalized strategies, including perioperative hydration, rigorous control of contrast volume, and criteria-based use of nephroprotective agents, are essential to minimize complications and optimize the safety of angiographic procedures.

## Data Availability

Os dados que sustentam este estudo estão disponíveis mediante solicitação à autora correspondente, J.P., devido a restrições éticas e de privacidade.

## Material Suplementar

Este artigo acompanha material suplementar.

STROBE Statement Checklist of items that should be included in reports of ***cohort studies***

Este material está disponível como parte da versão online do artigo na página https://doi.org/10.1590/1677-5449.202500281
