# Supplementary material for: Incidence and risk factors for contrast-induced nephropathy after angioplasty: an observational retrospective study
Source: J Vasc Bras. 2026 Jan 9;24:e20250028. doi: 10.1590/1677-5449.202500282 (PMC12822373; doi:10.1590/1677-5449.202500282)
Supplement: Translation

# Translation: Incidência e fatores de risco para nefropatia induzida por contraste após angioplastia: um estudo observacional retrospectivo

**DOI:** 10.1590/1677-5449.202500281

**Authors:** Juliana Peres, Jeferson Freitas Toregeani, Amanda Cristina Pohl, Ana Julia Vendrametto, Luciano de Andrade, André Brusamolin Moro

**Affiliations:** 1 Centro Universitário Fundação Assis Gurgacz – FAG, Cascavel, PR, Brasil.; 2 Universidade Estadual do Oeste do Paraná – UNIOESTE, Cascavel, PR, Brasil.; 3 Universidade Estadual de Maringá – UEM, Maringá, PR, Brasil.; 4 Hospital Estadual Vila Alpina, São Paulo, SP, Brasil.

**Keywords:** angioplastia, meios de contraste, injúria renal aguda, nefropatia, fatores de risco

## Abstract

**Contexto:**

A nefropatia induzida por contraste (NIC) é uma complicação do uso de contraste iodado, podendo levar à piora da função renal, ao aumento da morbimortalidade e à necessidade de terapia renal substitutiva.

**Objetivos:**

Avaliar a incidência de NIC após angioplastia e identificar fatores associados, incluindo variações na creatinina, na taxa de filtração glomerular, no volume de contraste, no tempo de procedimento e nas comorbidades.

**Métodos:**

Foi realizado um estudo retrospectivo com 305 pacientes submetidos à angioplastia. Foram analisadas variáveis clínicas e laboratoriais, incluindo creatinina sérica e taxa de filtração glomerular antes e após o procedimento. A NIC foi definida como o aumento absoluto da creatinina ≥ 0,5 mg/dL ou um aumento relativo ≥ 25% em até 72 horas. Aplicou-se regressão logística para identificar preditores independentes.

**Resultados:**

A incidência de NIC foi de 10,5% (n = 32). Pacientes com NIC apresentaram redução significativa da taxa de filtração glomerular (pré: 73,79 ± 22,5 mL/min *versus* pós: 34,32 ± 11,8 mL/min; p < 0,0001) e aumento da creatinina (pré: 1,12 ± 0,3 mL/min *versus* pós: 1,78 ± 0,6 mg/dL; p < 0,001). A NIC associou-se a acidente vascular encefálico (p = 0,014), doença arterial obstrutiva periférica (p = 0,007), diabetes melito (p = 0,002), doença renal crônica (p = 0,005) e insuficiência cardíaca (p = 0,004). A análise multivariada confirmou o diabetes melito (*odds ratio* = 2,45; IC95% 1,12-4,38; p = 0,022) como principal fator de risco.

**Conclusões:**

A NIC ocorreu em 10,5% dos pacientes, sendo diabetes melito, insuficiência renal crônica e insuficiência cardíaca os principais fatores de risco. Esses achados reforçam a importância do monitoramento para reduzir o impacto da NIC e otimizar desfechos clínicos.

## INTRODUÇÃO

A nefropatia induzida por contraste (NIC) representa uma complicação significativa em indivíduos submetidos a procedimentos de angioplastia, sendo frequentemente subdiagnosticada e subestimada^1^. A angiografia, uma técnica comum em cirurgia cardiovascular e vascular, permite a visualização das artérias e veias por meio de raios X, utilizando contrastes iodados para aprimorar as imagens vasculares^2,3^. Apesar dos avanços na formulação de meios de contraste de baixa osmolaridade, que são considerados menos deletérios^4,5^, a NIC permanece como a terceira principal causa de insuficiência renal aguda em ambientes hospitalares, afetando cerca de 10% dos doentes^6^. Esse quadro resulta em aumento de tempo de internação, necessidade de cuidados intensivos e elevação dos custos hospitalares com medicamentos e tratamentos dialíticos. Além disso, a NIC aumenta as taxas de readmissão e prolonga a recuperação do paciente, impactando diretamente na morbimortalidade^7^.

Embora a NIC seja uma preocupação crescente na prática clínica, ainda existem muitos fatores de risco que precisam ser explorados em profundidade, incluindo características epidemiológicas, duração da exposição ao contraste e comorbidades associadas^8^. A literatura atual apresenta uma variedade de dados e lições muitas vezes contraditórias sobre esses fatores, especialmente no que diz respeito ao limiar seguro de volume de contraste, à real influência de comorbidades associadas ao paciente na incidência de NIC e à efetividade das estratégias de hidratação na prevenção^4^. Embora o volume de contraste seja tradicionalmente associado à NIC^1,6,8^, estudos recentes demonstram resultados conflitantes, variando desde forte associação até ausência de relação causal^9,10^, destacando a necessidade de protocolos mais rigorosos para otimizar a dose utilizada. Esse cenário reforça a importância de uma investigação mais abrangente^11^.

A NIC é caracterizada pelas rápidas alterações da função renal após a administração de meios de contraste iodados, definindo-se pelo aumento absoluto da creatinina sérica ≥ 0,5 mg/dL ou por um aumento relativo ≥ 25% até 72 horas após a infusão de contraste^12-14^. A etiopatogenia da NIC é multifatorial, com a vasoconstrição figurando como um dos principais mecanismos, levando à isquemia medular renal e à redução da taxa de filtração glomerular (TFG)^2,6^. Além disso, os efeitos farmacotóxicos dos meios de contraste podem causar lesões celulares diretas, interferências tubulares e alterações osmóticas, provocando um efeito tóxico independente das alterações hemodinâmicas^15^. Os contrastes iodados podem provocar um aumento da concentração de sódio e da pressão osmótica no túbulo renal, contribuindo para a lesão tubular aguda e comprometendo ainda mais a função renal^16^.

Os fatores de risco para a NIC incluem condições preexistentes que prejudicam a função renal, como insuficiência renal crônica (IRC), diabetes melito (DM), idade avançada, insuficiência cardíaca (IC), dislipidemia e hipertensão arterial sistêmica (HAS)^17-19^. Além disso, características do meio de contraste, como o volume administrado e a osmolaridade, também influenciam no risco^13,20,21^, sendo os contrastes de alta osmolaridade mais propensos a aumentar a pressão osmótica e intensificar a vasoconstrição e a toxicidade renal^4,11^. Assim, a escolha do contraste deve considerar esses fatores para reduzir o risco de NIC.

A avaliação laboratorial é essencial para sujeitos submetidos ao procedimento de angioplastia com risco de desenvolver NIC, principalmente devido à complexidade da doença^11^. A avaliação da creatinina sérica é um dos principais métodos utilizados para detectar alterações na função renal. Estudos indicam que a elevação dos níveis de creatinina após a exposição ao contraste pode ser um preditor eficaz de lesão renal aguda, permitindo intervenções precoces e adequadas^22^. Contudo, pesquisas mais abrangentes indicam que o cálculo da TFG é um método mais preciso e confiável para avaliar a função renal. A TFG é essencial para identificar indivíduos com risco elevado de NIC, especialmente aqueles com IRC, DM e HAS. Esses fatores de risco estão fortemente associados ao desenvolvimento da NIC, reforçando a importância de uma avaliação renal prévia e contínua em pacientes que serão submetidos a procedimentos com contraste^23,24^. Além disso, marcadores laboratoriais adicionais são recomendados para uma avaliação completa da função renal^25^.

A monitorização laboratorial deve ser aliada a uma avaliação clínica abrangente, que leve em conta fatores como idade, comorbidades e o volume total de meio de contraste administrado em relação ao peso do paciente. A personalização do manejo, com base nas características de cada sujeito e nos resultados laboratoriais, é fundamental para reduzir o impacto da NIC e melhorar os desfechos clínicos^26^. As evidências apontam que a capacitação científica e a conscientização dos profissionais de saúde sobre os riscos dos contrastes iodados, juntamente com as avaliações laboratoriais rigorosas, são estratégias cruciais na prevenção da NIC^27,28^.

Objetivou-se investigar a incidência de NIC em pacientes submetidos a procedimentos de angioplastia em um hospital no Oeste do Paraná e identificar os fatores de risco associados, evidenciando a importância do acompanhamento clínico e laboratorial em um período mínimo de 48 horas.

## MÉTODOS

### Tipo e local de estudo

Este estudo observacional retrospectivo foi aprovado pelo Comitê de Ética em Pesquisa com Seres Humanos do Centro Universitário Fundação Assis Gurgacz (FAG), sob o Certificado de Apresentação de Apreciação Ética número 80063724.0.0000.5219, e número de parecer consubstanciado 6.949.781. Conduzido ao longo de 8 meses, objetivou analisar prontuários de doentes submetidos à angioplastia com uso de contraste em um hospital do Oeste do Paraná. Considerando sua natureza retrospectiva e a ampla população envolvida no estudo, houve dispensa do Termo de Consentimento Livre e Esclarecido, conforme aprovação ética.

### População do estudo

Este estudo utilizou o método de seleção consecutiva, incluindo todos os doentes submetidos à angioplastia com o uso de contraste iodado não iônico no período de 2021 a 2024, em um hospital escola no Oeste do Paraná. Para padronização, o mesmo meio de contraste intravenoso (Omnipaque®, Ioexol 300 mg/mL, 600-844 mOsm/Kg/H2O) foi utilizado em todos os casos.

Foram excluídos do estudo sujeitos com idade inferior a 18 anos, aqueles com exposição prévia ao contraste iodado nos últimos 30 dias anteriores à inclusão no estudo, indivíduos que evoluíram a óbito em menos de 48 horas após a administração do contraste, e aqueles que não possuíam dados pré ou pós-operatórios laboratoriais ou epidemiológicos suficientes para a análise.

Para minimizar o risco de seleção, os critérios de inclusão e exclusão foram padronizados, garantindo a representatividade da amostra sem interferências subjetivas. As exclusões foram limitadas a casos que poderiam comprometer a análise da NIC, evitando distorções nos resultados. A uniformização do meio de contraste eliminou variações na toxicidade renal, enquanto a definição objetiva da NIC impediu erros de categorização. A análise por regressão logística multivariada permitiu o controle de fatores de confusão, tornando a avaliação mais precisa e confiável.

No total, foram selecionados 353 indivíduos, dos quais 48 foram excluídos. Destes, 36 não possuíam exames laboratoriais pré ou pós-operatórios suficientes para indicar se houve NIC, dois evoluíram a óbito após as 24 horas seguintes à admissão hospitalar, e 10 já haviam passado por um procedimento com exposição a contraste iodado nos últimos 30 dias. Embora a amostra total do estudo fosse de 305 pacientes, foi possível obter dados completos sobre o volume de contraste em apenas 179 prontuários. A limitação na disponibilidade dessa variável ocorreu devido à ausência do registro do volume exato em parte dos prontuários eletrônicos, o que impossibilitou sua inclusão na análise estatística. Dessa forma, as avaliações que envolveram essa variável foram realizadas considerando exclusivamente os dados disponíveis, garantindo que as análises fossem conduzidas de forma transparente e sem viés de imputação (Figura 1).

**Figura 1:** Fluxograma do processo de seleção, exclusão e perdas dos participantes do estudo. NIC = nefropatia induzida por contraste.

Para garantir um poder estatístico adequado, calculou-se o tamanho amostral mínimo de 109 participantes, com base em uma fórmula para populações finitas apresentada na Equação 1 (considerando incidência de NIC = 12,5%, n = 305, IC95%, erro = 5%). Entretanto, optou-se por incluir todos os 305 casos elegíveis para aumentar a precisão das estimativas:


$$\begin{array}{c} \text{n}=\;\left[305\text{x}0,125\text{x}\left(1\;-\;0,124\right)\text{x}\left(1,96\right)²\right]\;/\;\\ \left\{\left[\left(305\;-\;1\right)\text{x}\left(0,05\right)²]\;+\;[0,125\text{x}\left(1\;-\;0,125\right)\text{x}\left(1,96\right)²\right]\right\} \end{array}$$ (1)

### Variáveis analisadas

As variáveis coletadas através dos prontuários eletrônicos foram: sexo, idade, peso, etnia, comorbidades preexistentes, níveis de creatinina sérica pré e pós-operatório (em até 72 horas após a realização do procedimento), procedimento realizado, volume de contraste utilizado e tempo de procedimento. As TFGs pré e pós-operatórias foram calculadas utilizando a equação de Cockcroft-Gault^29^.

### Desfecho principal

A NIC foi o desfecho principal analisado no estudo após procedimentos de angioplastia, tanto eletivos quanto emergenciais. A NIC é definida como rápida deterioração da função renal após a administração de meios de contraste iodados, sendo diagnosticada pelo aumento absoluto da creatinina sérica ≥ 0,5 mg/dL ou um aumento relativo ≥ 25% até 72 horas após a infusão do contraste^12,13^. O objetivo adicional do estudo foi avaliar a incidência de NIC e identificar fatores de risco associados, com especial atenção às comorbidades preexistentes dos pacientes.

### Análise de dados

Os dados coletados foram sintetizados e organizados em uma planilha de Excel® e, posteriormente, analisados na forma descritiva. As variáveis ​​contínuas (idade, peso, creatinina pré e pós-operatória, volume de contraste infundido e TFG pré e pós-operatória) foram apresentadas como média ± desvio padrão para distribuições normais, e como mediana e intervalo interquartil para distribuições não normais. As variáveis ​​categóricas foram expressas como frequências absolutas e percentuais. As comparações entre grupos (sujeitos com e sem NIC) para variáveis ​​contínuas foram realizadas utilizando o teste de Mann-Whitney (não paramétrico), por conta das distribuições não normais. As variáveis ​​categóricas foram comparadas utilizando o teste qui-quadrado ou o teste exato de Fisher, quando apropriado. Para identificar fatores associados à ocorrência de NIC, empregou‑se análise de regressão logística multivariada utilizando o método *backward stepwise* e a estatística de Wald. Foram apresentados os valores de *odds ratio* (OR) e IC95% para quantificar a magnitude das associações. Um valor de p < 0,05 foi considerado estatisticamente significativo em todas as análises.

Para as análises envolvendo o volume de contraste, foram aplicados os testes estatísticos não paramétricos apropriados, considerando sua distribuição não normal. A comparação entre os grupos com e sem NIC foi realizada através do teste de Mann-Whitney, enquanto a associação entre volume de contraste e tempo de procedimento foi avaliada por meio da correlação de Spearman. Além disso, a influência do tipo de procedimento sobre o volume de contraste foi analisada pelo teste de Kruskal-Wallis.

Todos os dados foram analisados ​​utilizando o *software* estatístico GraphPad Prism versão 10.3.9. A regressão logística *backward stepwise* foi utilizada para avaliar a correlação entre as variáveis independentes e o desenvolvimento de NIC, e a análise foi realizada utilizando o *software* Statistical Package for the Social Sciences® (versão 17.0 para Windows). Além disso, este estudo seguiu as recomendações do STROBE Statement para estudos observacionais, conforme apresentado no *Material Suplementar* – *STROBE Statement* disponibilizado junto à versão online do artigo.

## RESULTADOS

A amostra incluída no estudo foi de 305 indivíduos. Entre estes, havia 197 (64,6%) homens e 108 (35,4%) mulheres, sendo 190 pacientes brancos (62,3%) e 115 (37,7%) pardos e negros. A idade dos participantes variou de 28 a 90 anos, com média de 66,69 ± 11,02 anos e mediana de 68 anos. O peso variou entre 30 e 168 kg, com média de 76,52 ± 17,40 kg e mediana de 74 kg. A creatinina sérica pré-operatória apresentou média de 1,07 ± 0,54 mg/dL e mediana de 0,93 mg/dL, enquanto a creatinina pós-operatória apresentou média de 1,21 ± 0,77 mg/dL e mediana de 1,05 mg/dL. A TFG pré-operatória variou de 13,4 a 252,8 mL/min, com uma média de 80,2 ± 36,2 mL/min, e a TFG pós-operatória variou de 10,5 a 288,2 mL/min, com uma média de 74,0 ± 35,9 mL/min. O volume de contraste infundido variou de 2 a 600 mL, com média de 170 ± 99,0 mL e mediana de 150 mL. Após correção pela área de superfície corporal, encontrou-se o valor de 89,9 ± 52,4 mL/m^2^. A incidência de NIC foi de 10,5%.

Em relação às variáveis que abordaram o tempo médio de procedimento e a localização anatômica da angioplastia, aplicou-se o teste de Mann-Whitney, cujos resultados estão apresentados na Tabela 1.

**Tabela 1:** Local e tempo médio do procedimento.

**Localização**	**n total**	**Tempo médio**	**n com NIC**	**Tempo médio**
Angioplastia de coronárias	244	01:21 ± 00:48	20	01:34 ± 01:11
				p = 0,36
Angioplastia de artérias renais	2	01:41 ± 00:12	1	01:32
Tratamento endovascular das doenças da aorta	8	03:15 ± 00:34	2	03:16 ± 00:07
				p = 0,57
Angioplastia de carótida	25	01:54 ± 01:01	4	01:18 ± 00:22
				p = 0,37
Angioplastia de artéria subclávia	2	00:59 ± 00:36	1	00:33
Angioplastia de membros inferiores	23	01:42 ± 00:48	4	01:11 ± 00:37
				p = 0,19
Angioplastia de tronco supra aórtico	1	01:01	-	-
Total	305	01:28	32	01:33 ± 01:04
				p = 0,63

NIC = nefropatia induzida por contraste.

Fonte: dados da pesquisa.

Na comparação entre os sujeitos com e sem NIC, os que desenvolveram NIC apresentaram uma média de idade ligeiramente maior (69,5 anos *versus* 68,0 anos; p = 0,95). Em relação ao peso, também não houve diferença relevante entre os grupos (77,0 kg *versus* 74,0 kg; p = 0,98). Como esperado, observou-se uma tendência de redução da TFG pré-operatória nos indivíduos com NIC, comparativamente ao grupo sem NIC (61,08 mL/min *versus* 77,78 mL/min; p = 0,09), porém sem significância estatística. Contudo, a TFG pós-operatória foi significativamente mais baixa no grupo com NIC (34,32 mL/min *versus* 73,79 mL/min; p < 0,0001), o que indicou a deterioração da função renal após o procedimento nesses pacientes, como relatado no diagnóstico. Adicionalmente, a creatinina pré-operatória foi maior nos indivíduos com NIC (1,1 mg/dL *versus* 0,92 mg/dL; p = 0,03), e a elevação da creatinina foi ainda mais pronunciada no período pós-operatório, com valores médios de 2,1 mg/dL nos pacientes com NIC, em comparação a 1,00 mg/dL nos indivíduos sem NIC (p < 0,0001). As demais comparações de variáveis demográficas, clínicas e laboratoriais entre pacientes com e sem NIC não diferiram significativamente e são apresentadas na Tabela 2.

**Tabela 2:** Comparação de variáveis contínuas entre indivíduos com e sem NIC.

	**Sem NIC (n = 273)**	**Com NIC (n = 32)**	**Valor de p**
Idade (anos)	68.00 ± 11.00	69.50 ± 10.37	0.95
Peso (kg)	74.00 ± 17.31	77.00 ± 18.34	0.98
TFG pré-operatória (mL/min)	77.70 ± 35.46	61.08 ± 41.32	0.09
TFG pós-operatória (mL/min)	73.79 ± 34.95	34.32 ± 19.76	< 0.0001
Creatinina pré-operatória (mg/dL)	0.92 ± 0.40	1.10 ± 1.08	0.03
Creatinina pós-peratória (mg/dL)	1.00 ± 0.39	2.10 ± 1.50	< 0.0001

TFG = taxa de filtração glomerular; NIC = nefropatia induzida por contraste.

Fonte: dados da pesquisa.

A análise das comorbidades em relação à incidência de NIC (Tabela 3) revelou associações importantes entre certas condições clínicas e o aumento do risco de NIC. Comorbidades como acidente vascular cerebral (AVC), doença arterial obstrutiva periférica (DAOP), DM, IC e IRC apresentaram valores de p significativos (< 0,05), o que demonstrou que indivíduos com essas condições têm uma probabilidade maior de desenvolver a doença. O DM e a doença renal crônica (DRC) se destacaram, com OR de 3,1 e 20,77, respectivamente. Além disso, a IC apresentou OR de 3,1 (IC95% 1,2-8,0), reforçando seu papel no aumento do risco de NIC. Por outro lado, comorbidades como HAS e dislipidemia, apesar de serem frequentes entre os pacientes, não demonstraram associação significativa, conforme indicado pelos valores de p (> 0,05) e pela OR próxima de 1, o que indica que essas condições, quando isoladas, não aumentam o risco de NIC. No entanto, a prevalência de HAS entre os pacientes com NIC (71,88%) sugere que, embora não seja um fator de risco isolado, pode desempenhar um papel em conjunto com outras comorbidades.

**Tabela 3:** Comparação das comorbidades com incidência de NIC.

**Comorbidades**	**p (< 0,05)**	**OR**	**IC95%**
AVC	0.004	4.40	1.6-12.8
DAC	0.70	0.80	0.3-2.1
DAOP	0.02	3.40	1.2-10.1
Dislipidemia	0.14	0.50	0.2-1.1
DM	0.002	3.10	1.5-6.5
HAS	0.20	0.60	0.2-1.5
Hipotireoidismo	0.69	1.20	0.4-3.7
IAM	0.79	1.10	0.4-3.4
IC	0.01	3.10	1.2-8.0
DRC	< 0.0001	20.77	5.4-77.5
IVC	0.10	3.57	0.6-17.8
Tabagismo	0.10	1.80	0.8-3.8

NIC = nefropatia induzida por contraste; AVC = acidente vascular cerebral; DAC = doença arterial crônica; DAOP = doença arterial periférica obstrutiva; DM = diabetes melito; HAS = hipertensão arterial sistêmica; IAM = infarto agudo do miocárdio; IC = insuficiência cardíaca; DRC = doença renal crônica; IVC = insuficiência venosa crônica; OR = *odds ratio*.

Fonte: dados da pesquisa.

A análise do volume de contraste infundido entre os grupos com e sem NIC não evidenciou diferença estatisticamente significativa, conforme teste de Mann-Whitney. O grupo sem NIC recebeu em média 174 ± 115 mL, enquanto o grupo que desenvolveu NIC recebeu 183 ± 109 mL (p = 0,619), sugerindo que, isoladamente, o volume de contraste infundido não foi um fator determinante para a NIC na amostra.

Através da regressão logística *backward stepwise*, foram analisadas variáveis como sexo, idade, peso, etnia, tempo de procedimento, TFG antes e após o procedimento, além dos níveis de creatinina pré e pós-operatórios. Houve uma correlação positiva entre NIC e os valores de creatinina pós-operatória (p < 0,001, Exp(B) = 17,581), o que indicou um risco aumentado de NIC conforme o aumento da creatinina pós-operatória, como já observado na literatura. O valor Exp(B) representa a odds ratio estimada pelo modelo, ou seja, quantifica quanto aumenta (ou diminui) a chance de ocorrência do desfecho para cada unidade de aumento da variável preditora, mantendo as demais constantes. Nesse caso, o valor de 17,581 significa que, para cada aumento de 1 mg/dL na creatinina pós-operatória, a chance de desenvolver NIC foi aproximadamente 17,6 vezes maior. A Tabela 4 apresenta as variáveis do modelo de regressão logística durante o processo *backward stepwise*.

**Tabela 4:** Variáveis pela regressão de logística *backward stepwise*.

**Variável**	**Escore**	**df**	**Valor de p**
Sexo	4,828	1	0,028
Idade (anos)	1,079	1	0,299
Peso (kg)	0,001	1	0,974
Etnia	0,100	1	0,752
Tempo de procedimento (min)	0,188	1	0,664
TFG pré-operatória	20,570	1	< 0,001
Creatinina pré-operatória	63,530	1	< 0,001

TFG = taxa de filtração glomerular; df = graus de liberdade, ou seja, o número de valores independentes que podem variar na análise estatística.

Fonte: dados da pesquisa.

Em relação ao volume de contraste e tempo de procedimento, o teste de Spearman indicou uma correlação positiva, porém não significativa (ρ = 0,124; p = 0,098), sugerindo independência entre as variáveis. O teste de Kruskal-Wallis (qui-quadrado = 4,24; p = 0,644) e as comparações múltiplas de Dwass-Steel-Critchlow-Fligner (p > 0,05) não identificaram diferenças significativas no volume de contraste entre os tipos de angioplastia.

Na Tabela 5, o teste qui-quadrado avaliou a incidência de NIC estratificada por faixas de volume administrado e, embora o grupo > 200 mL apresentasse incidência numericamente maior (20,0% *versus* 10,1% no grupo ≤ 100 mL), essa diferença não foi estatisticamente significativa (p = 0,410). No entanto, a OR = 2,23 (IC95% 0,71-6,97) sugere uma possível relevância clínica, mesmo sem significância estatística.

**Tabela 5:** Análise da associação entre faixas de volume de contraste e NIC pelo teste qui-quadrado

**Faixa de volume infundido (mL)**	**n**	**Casos de NIC (%)**	**Risco relativo (IC95%)**	**Valor de p**
< 100	79	8 (10,1)	1,00 (Ref^*^)	-
101-200	70	8 (11,4)	1,13 (0,43-2,96)	0,814
> 200	30	6 (20,0)	1,98 (0,74-5,30)	0,108
Total	179	22 (12,3)		

* Valores de p calculados pelo teste exato de Fisher (bilateral) comparando cada faixa contra o grupo referência (≤ 100 mL). Um valor p < 0,05 foi considerado estatisticamente significativo. NIC = nefropatia induzida por contraste.

Fonte: dados da pesquisa.

## DISCUSSÃO

A análise revelou uma incidência de NIC de 10,5% em uma amostra de 305 pacientes submetidos a procedimentos de angioplastia. O achado está em concordância com a literatura existente, que aponta uma variação de incidência de 2 a 30%, dependendo das características dos pacientes e do tipo de contraste utilizado^13,30^. A observação de que a TFG pós-operatória foi significativamente reduzida em indivíduos que desenvolveram NIC (34,32 mL/min *versus* 73,79 mL/min; p < 0,0001) indica uma deterioração acentuada da função renal, evidenciando a gravidade da NIC, o que pode levar pacientes a um quadro de diálise e a um aumento da mortalidade nos casos de TFG ≤ 45 mL/min/1,73 m^29^ . Esses dados sugerem que a avaliação cuidadosa da função renal antes e após a administração de meio de contraste é crucial, especialmente em sujeitos com fatores de risco conhecidos, como DM e DRC^31,32^, que apresentaram OR de 3,1 e 20,77, respectivamente. Estes resultados corroboram a hipótese de que a função renal basal é um preditor significativo de NIC, reforçando a necessidade de monitoramento clínico intensivo nesse grupo de risco^33^.

A análise das comorbidades revelou que AVC, DAOP e IC também estão associados a um aumento significativo no risco de NIC, destacando a complexidade dos pacientes submetidos a intervenções angiográficas^10^. Notavelmente, a prevalência de HAS entre os pacientes com NIC (71,88%) sugere que, embora não tenha se mostrado um fator de risco isolado, pode interagir de maneira relevante com outras comorbidades, aumentando a vulnerabilidade ao desenvolvimento de NIC^11,34,35^. A interação entre múltiplas condições clínicas ressalta a necessidade de uma abordagem multidisciplinar para a avaliação de riscos e a implementação de estratégias de proteção renal, como a hidratação adequada e a utilização de agentes nefroprotetores quando indicado^6,13,27^.

Não houve diferenças estatisticamente significativas no tempo médio dos procedimentos entre os casos com e sem NIC para todas as localizações anatômicas, conforme o teste de Mann-Whitney (p > 0,05 para todas as comparações). O tempo médio dos procedimentos variou consideravelmente entre as diferentes localizações, sendo mais longo para tratamentos endovasculares de doenças da aorta. A ausência de significância estatística sugere que o tempo do procedimento, isoladamente, pode não ser um fator determinante para o desenvolvimento de NIC. No entanto, a análise está sujeita a vieses, como o pequeno número de procedimentos em certas localizações (por exemplo, angioplastia de artérias renais e tronco supra-aórtico), o que limita a precisão das comparações^36^.

A regressão logística *backward stepwise* identificou a creatinina pós-operatória como o principal marcador de NIC, com uma OR de 17,5, reafirmando estudos anteriores que enfatizam a importância do monitoramento da creatinina após a administração de contraste em até 48 horas após o procedimento^37,38^. A correlação positiva entre os níveis elevados de creatinina pós-operatória e o desenvolvimento de NIC é um indicativo claro de que a intervenção precoce em pacientes com alterações na função renal pode ser vital para prevenir complicações adicionais^13^. Esses resultados reforçam a necessidade de protocolos clínicos que incluam a avaliação da função renal em tempo real durante e após os procedimentos de angioplastia, visando a identificação precoce e a intervenção oportuna em pacientes em risco, com o intuito de minimizar a morbidade associada à NIC^8,32,39,40^. A partir das implicações clínicas discutidas, é evidente que a personalização das abordagens terapêuticas em pacientes submetidos à angioplastia deve ser baseada em uma compreensão aprofundada das comorbidades e dos fatores de risco individuais.

Apesar de o volume de contraste ser amplamente reconhecido como fator de risco para NIC, neste estudo, não foi encontrada uma associação estatisticamente significativa entre essa variável e o desfecho renal. Esse achado contrasta com estudos anteriores que demonstram uma relação dose-dependente entre o volume de contraste e a função renal^11,13^. No entanto, essa discrepância pode ser explicada por alguns fatores metodológicos e clínicos.

Primeiramente, a disponibilidade limitada de informações sobre o volume de contraste em nossa amostra (apenas 172 dos 305 pacientes) reduziu o poder estatístico da análise, dificultando a identificação de uma possível associação. Além disso, práticas clínicas como hidratação perioperatória adequada e o uso preferencial de contrastes de menor osmolaridade podem ter mitigado os efeitos nefrotóxicos do contraste, reduzindo o impacto sobre a função renal^6,27,34^. Evidências sugerem que a hidratação guiada por estratégias individualizadas pode ser mais eficaz na prevenção da NIC do que a simples restrição do volume de contraste^35^.

Outro ponto relevante é que a NIC é um fenômeno multifatorial, influenciada por condições preexistentes, como DM, IRC e IC, fatores que se mostraram significativamente associados à ocorrência de NIC em nossa análise. Estudos prévios demonstram que a predisposição individual desempenha um papel mais determinante do que o volume de contraste isoladamente, especialmente em pacientes com TFG reduzida^15,19^. Isso pode explicar por que, mesmo em estudos que demonstram associação entre volume de contraste e a NIC, os limiares de risco variam amplamente dependendo das características dos pacientes^16^.

Este estudo apresenta algumas limitações que devem ser consideradas. A amostra, embora representativa, pode não refletir completamente a população submetida à angioplastia, limitando a generalização dos achados. Além disso, a análise foi conduzida em um único centro, o que pode introduzir vieses relacionados às práticas institucionais e ao perfil específico dos pacientes atendidos. O delineamento retrospectivo impõe desafios, tais como possíveis imprecisões nos registros médicos e a ausência de informações planejadas sobre fatores de risco adicionais ou estratégias de proteção renal^7^.

Outra limitação relevante diz respeito à ausência de informações sobre o uso de medidas nefroprotetoras, como estatinas e N-acetilcisteína, que podem ter influenciado os desfechos renais^5,18^. Além disso, a variabilidade na abordagem terapêutica, incluindo o tempo de hidratação pré e pós-procedimento, pode ter contribuído para a heterogeneidade dos resultados.

Dessa forma, estudos futuros são necessários para melhor esclarecer a relação entre volume de contraste e NIC. Pesquisas com delineamento prospectivo, inclusão de grupos de controle e critérios diagnósticos mais abrangentes para lesão renal podem fornecer evidências mais robustas sobre o impacto do volume de contraste na função renal e identificar subgrupos de pacientes com maior vulnerabilidade a esse efeito^20,24^.

## CONCLUSÃO

A monitorização rigorosa da função renal e a avaliação das comorbidades são fundamentais para reduzir o risco de NIC e melhorar os desfechos clínicos em pacientes submetidos à angioplastia. No presente estudo, DRC e DM foram identificados como fatores de risco significativos, destacando a necessidade de protocolos específicos para os pacientes com essas condições. A elevada OR associada à DRC reforça sua relevância como determinante crítico da NIC, exigindo precauções adicionais para minimizar o dano renal. Do mesmo modo, a associação significativa entre DM e NIC enfatiza a interação entre disfunção metabólica e vulnerabilidade renal, justificando abordagens preventivas diferenciadas para esse subgrupo. Dessa forma, estratégias personalizadas, incluindo hidratação perioperatória, controle rigoroso do volume de contraste e uso criterioso de agentes nefroprotetores, são essenciais para minimizar complicações e otimizar a segurança dos procedimentos angiográficos.

## Data Availability

The data supporting this study are available upon request from the corresponding author, J.P., due to ethical and privacy restrictions.

## Supplementary Material

Supplementary material accompanies this paper.

STROBE Statement Checklist of items that should be included in reports of ***cohort studies***.

This material is available as part of the online article from https://doi.org/10.1590/1677-5449.202500282
